# Microbial Community Analysis of a Methane-Producing Biocathode in a Bioelectrochemical System

**DOI:** 10.1155/2013/481784

**Published:** 2013-09-25

**Authors:** Mieke C. A. A. Van Eerten-Jansen, Anna B. Veldhoen, Caroline M. Plugge, Alfons J. M. Stams, Cees J. N. Buisman, Annemiek Ter Heijne

**Affiliations:** ^1^Sub-Department of Environmental Technology, Wageningen University, Bornse Weilanden 9, 6700 AA Wageningen, The Netherlands; ^2^Laboratory of Microbiology, Wageningen University, Dreijenplein 10, 6703 HB Wageningen, The Netherlands

## Abstract

A methane-producing biocathode that converts CO_2_ into methane was studied electrochemically and microbiologically. The biocathode produced methane at a maximum rate of 5.1 L CH_4_/m^2^ projected cathode per day (1.6 A/m^2^) at −0.7 V versus NHE cathode potential and 3.0 L CH_4_/m^2^ projected cathode per day (0.9 A/m^2^) at −0.6 V versus NHE cathode potential. The microbial community at the biocathode was dominated by three phylotypes of Archaea and six phylotypes of bacteria. The Archaeal phylotypes were most closely related to *Methanobacterium palustre* and *Methanobacterium aarhusense*. Besides methanogenic Archaea, bacteria seemed to be associated with methane production, producing hydrogen as an intermediate. Biomass density varied greatly with part of the carbon electrode covered with a dense biofilm, while only clusters of cells were found on other parts. Based on our results, we discuss how inoculum enrichment and changing operational conditions may help to increase biomass density and to select for microorganisms that produce methane.

## 1. Introduction

In bioelectrochemical systems (BES), microorganisms catalyze oxidation and reduction reactions to produce or use electricity. Recently, it has been discovered that microorganisms can accept electrons from an electrode [1] to bioremediate metal and organic contaminants or for microbial electrosynthesis to produce fuels and chemicals. Using microorganisms as catalysts on an electrode instead of chemical catalysts is innovative and sustainable; the microorganisms are self-regenerating and the BES can be operated at ambient conditions (at neutral pH and low temperature), and low-cost carbon electrodes can be used [2, 3]. Microbial electrosynthesis in BES has been described for the production of, for example, hydrogen [4], hydrogen peroxide [5], caustic [6], acetate and 2-oxobutyrate [7, 8], ethanol [9], ammonium [10], butyrate [11], or caproate and caprylate [12]. 

Another attractive application of microbial electrosynthesis is the conversion of CO_2_ into methane [13]. Besides producing carbon-neutral methane, BES can convert excess renewable electricity from sun and wind into methane as an energy carrier [13]. Moreover, the infrastructure for transport, storage and consumption of methane is already in place [13].

To improve the performance of a methane-producing BES, focus so far was mainly on BES design [14–17]. However, another key challenge is understanding the methane-producing microbial communities in order to improve the methane production rate and energy efficiency [18]. The microbial consortium (types of microorganisms, community composition, and interaction between microorganisms) and the biomass density (the number of microorganisms at a specified electrode surface or reactor volume that take part in these processes) will influence the performance of methane-producing biocathodes [18]. Selecting for electrochemically active microorganisms that produce methane and operating the BES under the most favorable conditions for the selected microorganisms may help to further improve the performance of a methane-producing BES [18]. 

The microbial composition of a methane-producing biocathode using enriched cultures as inoculum is scarcely documented [8, 13]. Cheng and coworkers obtained an enriched mixed-culture methane-producing biocathode after inoculating the cathode with the effluent of an existing bioanode [13]. Therefore, it was known beforehand that electrochemically active microorganisms would be present in the biofilm. The methane-producing biocathode consisted of an enriched consortium dominated by *Methanobacterium palustre*, which accounted for 86% of the total number of cells. A biocathode inoculated with a pure culture of *M. palustre*, however, produced less methane than the mixed-culture biocathode [13]. The role of the other detected microbial community members in methane production was not investigated. Marshall and co-workers obtained an enriched mixed-culture methane-producing biocathode after inoculating the cathode with brewery wastewater sludge that was pretreated at −0.59 V versus NHE cathode potential and that produced methane at this potential [8]. The microbial community at the methane-producing biocathode consisted of methanogens related to *Methanobacterium* sp. (>93%) and *Methanobrevibacter *(~5%) and bacteria related to the *Sphingobacteriales* WCHB1-69 family (37.7%), the *Spirochaetaceae* family (17.4%), and the *Synergistaceae* family (11.1%) [8]. It is possible that the bacteria related to the *Sphingobacteriales* family catalyzed bioelectrochemical hydrogen production [8], but the role of the other bacteria was not investigated. 

Although the microbial community of methane-producing biocathodes has been described before, the possible roles of the detected community members in methane production remain unclear. In this study, the electrochemical performance and microbial community of a mixed-culture methane-producing biocathode were investigated to illuminate the possible role of the detected community members in methane production. 

## 2. Materials and Methods

### 2.1. Electrochemical Cell

**Figure 1:**
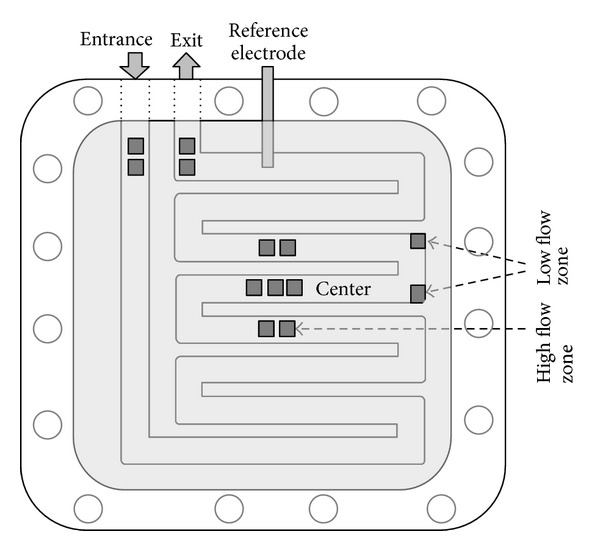
Schematic representation of the cathode chamber with the locations of the samples (dark grey squares) used for microbiological analysis. Samples were about 1 × 1 cm^2^. The high flow zone is where the cathode was in contact with the straight part of the flow channels, while the low flow zone is where the cathode was located in a dead zone. The graphite felt electrode (light grey) was placed between two supportive flow channel plates. The total projected surface area of cathode was 361 cm^2^, while the effective geometric channel surface area was 290 cm^2^.

A flat plate electrochemical cell (1.24 L total volume) was used with a cathode and anode volume of 0.62 L each (described in more detail in [19]), using a cation exchange membrane (fumasep FKB, FuMA-Tech GmbH, Germany). Both the anode and cathode were made of graphite felt (19 × 19 cm, thickness 3 mm FMI Composites Ltd., Scotland) having an effective geometric channel surface area of 290 cm^2^ each. The electrolytes flowed parallel to the electrodes through serpentine flow channels in both the anode and cathode compartments (Figure 1). The anode and cathode compartments were equipped with an Ag/AgCl, 3 M KCl reference electrode (+0.205 V versus NHE; ProSense QiS, The Netherlands). The reference electrode was connected to a glass capillary with a membrane tip that was inserted at the top of the cathode near the outlet (the glass capillary was positioned 5 mm from the graphite felt; Figure 1). The cathode headspace was connected to a gas flow meter (MilliGascounter, Ritter, Germany) via an injection port containing a septum. The cathode headspace volume was on average of 750 ± 250 mL and varied due to the batch-wise feeding of the catholyte. The experiment was performed in a temperature controlled chamber at 30°C.

### 2.2. Electrolytes and Microorganisms

The anolyte consisted of 50 mM potassium hexacyanoferrate(II) in deionized water. The anolyte was recirculated over a 10 L vessel at 1.5 mL/s and replenished on days 16, 28, and 49 to avoid depletion of electron donor. The catholyte consisted of 20 mM potassium phosphate buffer, macronutrients (280 mg/L NH_4_Cl, 5.7 mg/L CaCl_2_, 10 mg/L MgSO_4_·7H_2_O, and 90 mg/L MgCl_2_·6H_2_O), 1 mL/L of a micronutrients solution, and 1 mL/L of a vitamin solution as described in [20]. To the catholyte 5 g/L NaHCO_3_ was added as a carbon source, since at the operating conditions of pH 7, CO_2_ is predominantly present as bicarbonate. The catholyte was recirculated over a 0.5 L vessel at 1.5 mL/s. To avoid depletion of substrate, every two to three days 250–350 mL catholyte in the electrochemical cell was replaced by 250 mL fresh catholyte under continuous flushing with nitrogen gas. The cathode was inoculated with 5 grams of anaerobic sludge obtained from an upflow anaerobic sludge blanket reactor treating distillery wastewater (Royal Nedalco, The Netherlands). After inoculation, the system was flushed with pure nitrogen (>99.9992%) for 30 minutes before applying a cell voltage and starting the experiment. The pH of the catholyte was controlled at pH 7 ± 0.1 by a pH controller (Liquisys M CPM 253, Endress + Hauser, Switzerland) using 1 M HCl.

### 2.3. Electrochemical Cell Operation

The electrochemical cell was connected to a potentiostat (MCP94, Bank Elektronik Intelligent Controls GmbH, Germany) using as a two-electrode configuration where the cathode was connected to the work electrode and the anode was connected to both the counter electrode and the reference electrode. From the beginning, the electrochemical cell was operated at cathode potential −0.7 V versus NHE, as at this cathode potential methane can be both produced directly or via hydrogen as an intermediate [21]. From the moment that only methane and no hydrogen were detected in the cathode gas phase (day 24), the cathode potential was changed to −0.6 V versus NHE to decrease the energy input of the electrochemical cell. The cathode potential was controlled via the cell voltage as described in [17]. The cell voltage was adjusted when the cathode potential deviated >20 mV from the desired cathode potential. The experiment was terminated on day 57, due to leakages at the anode.

### 2.4. Analysis and Calculations

The electrochemical cell was connected to a PC via a Fieldpoint FP-AI-110 module (National Instruments, USA), and every 60 seconds cell voltage, current, and cathode and anode potential were measured using LabVIEW 7.1 (National Instruments, USA). Daily averages were calculated and reported throughout this paper.

Gas composition of the cathode gas phase was measured on days 0, 12, 24, 33, 51, and 57 with two different gas chromatographs to measure methane, hydrogen, and oxygen. Gas samples were taken with a 100 *μ*L gastight syringe (Hamilton, USA) from the injection port near the gas flow meter. Hydrogen was measured with an HP 5890A gas chromatograph by injecting 100 *μ*L of gas-sample on a molsieve column (30 m × 0.53 mm × 0.25 mm) with thermal conductivity detection (TCD). The oven temperature was 40°C, the injection gate was 110°C, and the TCD was 150°C. The carrier gas was argon and had a flow rate of 20 mL/min. Methane and oxygen were measured with a Finsons Instruments GC 8340 gas chromatograph. Gas (100 *μ*L) was split (1 : 1) over a molsieve column (30 m × 0.53 mm × 25 mm) and a PoraBOND Q column (25 m × 0.53 mm × 10 mm). The oven temperature was 40°C, injection gate was 110°C, and the TCD was 90°C. The carrier gas was helium and had a flow rate of 45 mL/min. Gas composition was measured immediately after replenishing the catholyte and just before the next catholyte replenishment. The time between catholyte replenishments was two to three days. Gas production was continuously measured with a gas flow meter (Milligascounter, Ritter, Germany). Methane production was calculated using the total gas production and the measured methane fractions, as in [22].

To compare methane production rates with those reported in the literature, all rates were calculated at standard temperature and pressure (STP, 273.15 K and 1 atm) and with respect to the projected cathode surface area (1) or total reactor volume (2) according to

(1) rCH4STP=VCH4,t·TSTP·pACat·Δt·T·pSTP,

(2) rCH4STP=VCH4,t·TSTP·pVreactor·Δt·T·pSTP,

where *r*_CH_4__^STP^ is the methane production rate at STP (L CH_4_/m^2^ projected cathode per day or L CH_4_/L reactor per day), *V*_CH_4_,*t*_ is the cumulative methane gas production (L) on sample time *t*, *A*_cat_ is the projected cathode surface area (0.029 m^2^), *V*_reactor_ is the total reactor volume (1.24 L), *t* is the time (s), *T* is the temperature used in this study (303 K), *p* is the pressure used in this study (1.005 atm), and *T*_STP_ and *p*_STP_ are the temperature and pressure at STP, 273.15 K and 1 atm, respectively. Cathodic electron efficiency, the efficiency of capturing the electrons from the electric current in methane, was calculated as in [17]:

(3) $$\begin{array}{c} \eta_{\text{CE}}=\frac{V_{\text{C}{\text{H}}_{4},t}\cdot F\cdot n}{V_{m}\cdot \int_{t=0}^{t}I\;dt}\cdot 100\%, \end{array}$$

where *F* is faraday constant (96485 C/mole e^−^), *n* is the moles of electrons per mole of methane (8 mole e^−^/mole CH_4_), *V*_*m*_ is the molar volume (22.7 L/mole at 273.15 K and 1 atm), *I* is the current (A), and *t* is the time (s).

### 2.5. Microbiological Characterization of the Methane-Producing Biocathode

At the end of the experiment (day 57), the cathode was cut into samples of about 1 × 1 cm^2^. These samples were used to characterize the microorganisms that had developed at the methane-producing biocathode. The samples were taken at three different locations at the cathode: where the catholyte entered the electrochemical cell (referred to as “entrance”), the center of the cathode (referred to as “center”), and where the catholyte left the electrochemical cell (referred to as “exit”). The locations of the samples are indicated in Figure 1.

The microorganisms present at the biocathode were identified using the molecular techniques described below. The amount of volatile suspended solids (VSS) was quantified using the modified Hartree-Lowry protein analysis. The morphology and distribution of microorganisms at the biocathode were visualized by fluorescence microscopy and scanning electron microscopy. 

#### 2.5.1. Microbial Community Analysis

Community analysis was performed at Nadicom GmbH Microbiology Services (Germany). Total genomic DNA was extracted from the 1 × 1 cm^2^ cathode sample taken in the high flow zone (Figure 1) of the center of the biocathode using the DNA extraction kit from AppliChem (Germany) according to manufacturer's instructions. PCR amplification of the bacterial 16S rRNA gene was performed corresponding to standard operating procedure (SOP) AD-01, using primers 27f and 1492r [23]. PCR protocols for amplification were initial denaturation for 5 minutes at 95°C, followed by 28 cycles of denaturation (20 seconds at 94°C), annealing (30 seconds at 55°C), and extension (60 seconds at 72°C), followed by a final extension (10 minutes at 72°C). The amplicons were stored at 8°C until further analysis. For the identification of methanogenic Archaea (indicated as “methanogens” in the rest of the manuscript), PCR was performed corresponding to SOP AD-01-1 [24], using primers Ar109f and Ar907r to amplify archaeal 16S rRNA. PCR protocols for amplification were initial denaturation for 5 minutes at 94°C, followed by 28 cycles of denaturation (60 seconds at 94°C), annealing (60 seconds at 52°C), and extension (90 seconds at 72°C), followed by a final extension (6 minutes at 72°C) [24]. The samples were stored at 4°C until further analysis. Archaeal and bacterial PCR amplicons were purified with the ChargeSwitch PCR Clean-Up Kit (Invitrogen, USA) according to manufacturer's instructions and cloned into *E. coli* JM109 using the TOPO TA Cloning Kit (Invitrogen, USA). After blue/white screening, positive colonies were transferred to LB medium containing 100 *μ*g/mL ampicillin and were grown overnight at 37°C. Plasmid DNA was isolated using the PureLink Quick Plasmid Miniprep Kit (Invitrogen, USA) according to the manufacturer's instructions. The PCR product quality was checked by agarose-gel-electrophoresis (1%) stained with ethidium bromide. PCR products with a size of 1.7 Kb were screened with a specific digestion using enzyme *MSP*1. Clones showing a unique band pattern were sequenced via cycle sequencing. The obtained sequences were compared to reference sequences in the NCBI BLAST database (http://blast.ncbi.nlm.nih.gov/). A phylogenetic classification was obtained, together with the degree of similarity to the reference sequences. Sequences retrieved in this study are accessible in the GenBank database under the accession numbers KC821541-KC821549.

#### 2.5.2. Modified Hartree-Lowry Analysis

The modified Hartree-Lowry method was used to determine the protein concentration per m^2^ of biocathode in order to quantify the biomass density (expressed as volatile suspended solids (VSS) per m^2^ projected cathode surface area). The modified Hartree-Lowry method was applied to two-entrance samples, two-center samples, and two-exit samples to investigate the effect of location on microbial cell concentration (Figure 1). For all samples, the exact surface area of the sample was measured. Each sample was transferred in a 2 mL vial, suspended in 1 mL 1 M NaOH, and mixed vigorously for 30 seconds to make sure biomass was suspended and not attached to the graphite felt. The vial was left at 46°C for 35 min to hydrolyze the cells, and the sample was subsequently neutralized with 1 mL 1 M HCl. The samples were once again mixed vigorously for 30 seconds. The suspension was filtered over a 2 *μ*m filter paper (Whatman 589/3, GE Healthcare, UK) to separate hydrolyzed cells from graphite fibers. To 0.5 mL filtrate, 2.5 mL filtered Lowry medium (19.6 g/L Na_2_CO_3_, 0.20 g/L Na_3_C_6_H_5_O_7_, and 0.1 g/L CuSO_4_·5H_2_O) was added, and the solution was mixed vigorously. After 15 minutes, 0.25 mL Folin-Ciocalteu's phenol reagent was added to the solution, and again the solution was mixed vigorously. After 25 minutes, the solution was spectrophotometrically analyzed at 650 nm (XION 500 spectrophotometer, Hach Lange GmbH Germany). The biomass density (g VSS/m^2^ projected cathode surface area) was calculated according to

(4) ρVSS=Cprotein·Vspec·4  0.25·Afelt  ,

where *C*_protein_ is the spectrophotometrically analyzed protein concentration derived from calibration measurements in which bovine serum albumin was used as reference protein (g/mL), *V*_spec_ is the total sample volume that was spectrophotometrically analyzed (3.25 mL), 4 is the dilution factor of the original sample, 0.25 is the conversion of g protein to g VSS, and *A*_felt_ is the surface area of the biocathode sample (m^2^).

#### 2.5.3. Fluorescence Microscopy

Methanogenic archaea have a low-potential electron carrier coenzyme F_420_ that emits a blue-green autofluorescence when exposed to ultraviolet light at a wavelength of 420 nm. Therefore, immediately after dismantling the methane-producing BES (day 57), two samples of the high flow zone of the center of the biocathode (Figure 1) were observed under a UV fluorescence microscope (Leica DMR FC4 microscope with Leica DFC340 FX camera, Germany) with filter cube I3 to identify the presence of active methanogens. The 3D structure and the 3 mm thickness of the graphite felt electrode made it impossible to observe the intact biocathode under the UV fluorescence microscope. Therefore, graphite fibers were taken from the graphite felt electrode and were observed under the UV fluorescence microscope.

#### 2.5.4. Scanning Electron Microscopy

Two samples from a low flow zone (where the cathode was located in a dead zone) and two samples of a high flow zone (where the cathode was in contact with the straight part of the flow channels) at the center of the biocathode (Figure 1) were analyzed using scanning electron microscopy. The biocathode samples were fixed for 2 hours in 2.5% glutaraldehyde in PBS (130 mM NaCl in 10 mM phosphate buffer pH 7.2). After fixation, the samples were washed with PBS for 3 times for 15 minutes per washing step. The samples were dehydrated through a series of ethanol baths of increasing concentrations: 10, 25, 50, 75, and 90% (v/v), 20 minutes each, and finally in 100% (v/v) ethanol for 30 minutes. The samples were dried in a desiccator and finally sputter coated with a 5 nm thin gold layer before visualization under high vacuum with a JSM-6480 LV scanning electron microscope (Jeol, Japan) at 10 kV accelerating voltage.

## 3. Results and Discussion

### 3.1. Performance of the Methane-Producing Biocathode

**Figure 2:**
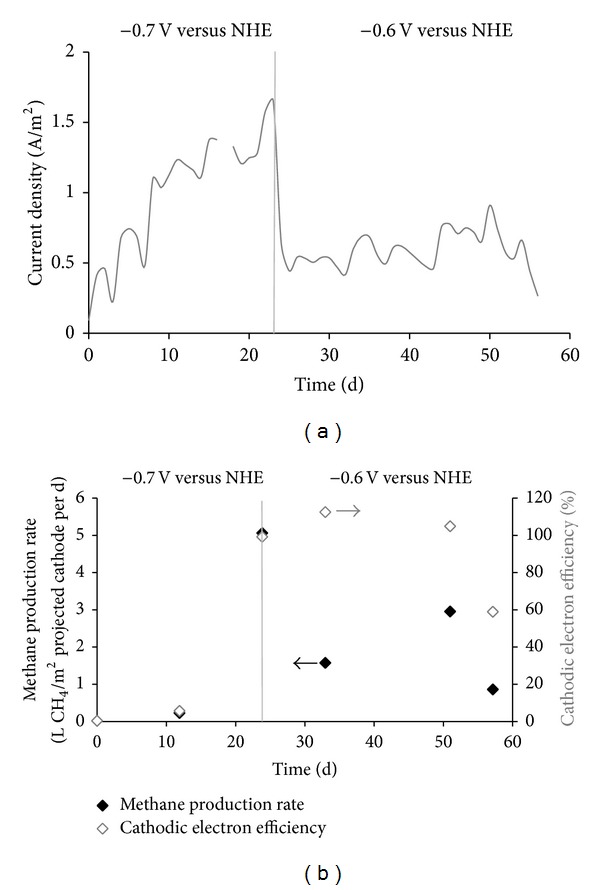
(a) Current density (daily averages) and (b) methane production rate and cathodic electron efficiency with time. Cathode potential was changed from −0.7 V versus NHE to −0.6 V versus NHE (day 24), as indicated by the grey vertical line.

Current consumption started directly after applying a cathode potential at the start of the experiment. Current density increased to 1.6 A/m^2^ projected cathode (day 24, Figure 2(a)). On day 24, solely methane and no hydrogen were detected in the cathode gas phase, indicating an active methane-producing biocathode. On day 24, the cathode potential was changed from −0.7 V to −0.6 V versus NHE. After changing the cathode potential, current density was rather constant, with a daily average of 0.60 ± 0.16 A/m^2^ projected cathode. 

Along with electric current consumption, hydrogen and methane were produced at the cathode. On day 12, only hydrogen (35.7% H_2_ (v/v)) was detected in the cathode headspace. On day 24, only methane was detected in the cathode headspace (29.5% CH_4_ (v/v)). Maximum methane production rate was 5.1 L CH_4_/m^2^ projected cathode per day (119 mL CH_4_/L reactor per day at standard temperature and pressure, STP) at −0.7 V versus NHE cathode potential (1.6 A/m^2^, day 24; Figure 2(b)). Maximum methane production rate was 3.0 L CH_4_/m^2^  projected cathode per day (69 mL CH_4_/L reactor per day, at STP) at −0.6 V versus NHE cathode potential (0.9 A/m^2^, day 51; Figure 2(b)).

Cathodic electron efficiency, the efficiency of capturing the electrons from the electric current in methane, increased from the start of the experiment from 0% (day 0) to 6% (day 12) to 99% (day 24) at −0.7 V versus NHE cathode potential. If the hydrogen produced at day 12 was included, the cathodic electron efficiency would increase to 49%, assuming 4 moles of hydrogen are required per mole of methane. After changing the cathode potential to −0.6 V versus NHE, cathodic electron efficiency was 92 ± 29% (average of days: 33, 51, and 57) (Figure 2(b)). Cathodic efficiencies of >100% have been reported previously [14, 17] and have been explained by biomass degradation and oxidation [17] or oxidation of carbon stored inside the biomass [25].

Reported methane production rates for methane-producing biocathodes in BES are between 0.12 and 24 L CH_4_/m^2^ projected cathode per day (0.07 to 15 A/m^2^) at ≤−0.55 V versus NHE cathode potential (Table 1). The methane production rates and current densities of the biocathode in this study were in the range of reported methane production rates and current densities. Nearly all of the reported studies used undefined enriched or mixed cultures as inoculum for the methane-producing biocathode. In these studies, the microbial populations were not analyzed. Therefore, it is not clear how the microbial community composition affected the performance of the methane-producing biocathode, and via which mechanisms methane was produced. To optimize methane production in BES, key challenges are to select for microorganisms that produce methane at high rate and to operate the BES under their most favorable conditions [18]. This study therefore investigated the microbial community at a methane-producing biocathode and their possible role in bioelectrochemical methane production. 

### 3.2. Characterization of the Methane-Producing Microbial Community at the Biocathode

Samples from the biocathode were used to characterize the composition of the microbial community. The microorganisms that were present at the center of the methane-producing biocathode are reported in Table 2. Three phylotypes of archaea and six phylotypes of bacteria were identified in the methane-producing biocathode. 

Archaeal 16S rRNA gene sequences were similar to the hydrogen-consuming *Methanobacterium palustre* strain DSM 3108 (98%, KC821542 and KC821543) and the hydrogen-consuming *Methanobacterium aarhusense *strain H2-LR (96%, KC821541).

 Bacterial 16S rRNA gene sequences were similar to *Methylocystis *sp. SC2 strain SC2 (98%, KC821549), *Acidovorax caeni *strain R-24608 or *Hydrogenophaga caeni* strain EMB71 (98%, KC821548), *Desulfovibrio putealis *strain B7-43 (97%, KC821546), *Petrimonas sulfuriphila *strain BN3 (96%, KC821544; 95%, KC821545), and *Ottowia thiooxydans *(95%, KC821547).

### 3.3. Possible Role of Microorganisms in Bioelectrochemical Methane Production

The methane-producing biocathode analyzed in this study contained three phylotypes of archaea: two phylotypes were closely related to *Methanobacterium palustre,* and the other phylotype was related to *Methanobacterium aarhusense*. *Methanobacterium palustre* can use hydrogen as an electron donor [26], although direct use of the electrode as electron donor has also been suggested [13]. *Methanobacterium palustre* has previously been identified as the dominant microorganism, accounting for 86% of the total cells, in a mixed-culture methane-producing biocathode inoculated with effluent of a bio-anode that was fed acetate [13]. *Methanobacterium aarhusense* can only use hydrogen as electron donor [27]. Bioelectrochemical production of hydrogen has been reported previously at the cathode potential used in this study [28]. It is likely that the phylotypes that were closely related to *M. palustre* and *M. aarhusense *used hydrogen as electron donor for bioelectrochemical production of methane. While at the start of the experiment hydrogen was detected in the cathode gas phase, no hydrogen was detected in the cathode gas phase once the biocathode had developed. In the experimental setup, it could not be distinguished whether *M. palustre* also used the electrode as electron donor. 

The methane-producing biocathode contained six phylotypes of bacteria. Bacteria identified in our biocathode that may be associated with bioelectrochemical production of methane were closely related to *Desulfovibrio putealis*, *Hydrogenophaga caeni*, and *Methylocystis *sp..* Desulfovibrio putealis* is a strict anaerobic microorganism that is able to use hydrogen, organic acids, or alcohol as an electron donor and sulfate as an electron acceptor [29]. However, it can only grow with hydrogen as electron donor when acetate is provided as carbon source [29]. Several *Desulfovibrio *sp. are able to catalyze bioelectrochemical hydrogen production at cathode potentials ≤−0.44 V versus NHE (e.g., [2, 3]). In study, the applied cathode potential was ≤−0.6 V versus NHE, being in the range of applied potentials at which *Desulfovibrio* sp. is reported to be electrochemically active. Therefore, we hypothesize that the phylotype that is closely related to *D. putealis* may have produced hydrogen, which in turn could be consumed by the methanogens to produce methane. Future research could focus on bioelectrochemical production of methane by *M. palustre* in the presence and absence of *D. putealis* in order to identify the role of the latter.

*Hydrogenophaga caeni* is an aerobic microorganism that is able to use hydrogen as an electron donor, however, only when an organic carbon source is provided [30, 31]. *Hydrogenophaga* sp. have also been found at hydrogen-producing biocathodes [32], but their role in hydrogen production remains unclear. The phylotype that is closely related to *Hydrogenophaga caeni* may have catalyzed hydrogen production or may have been an oxygen scavenger, creating strict anoxic conditions that are essential for the methanogenic archaea. 

*Methylocystis *sp. is a facultative aerobic microorganism that is able to use methane as the sole source of carbon and energy [33]. The phylotype that shows similarity to *Methylocystis *sp. may have consumed part of the methane, thereby lowering the methane production rate. However, as oxygen scavenger, it will also create the anoxic conditions that are essential for proliferation of methanogenic archaea. Another phylotype that may have been an oxygen scavenger is closely related to *Acidovorax caeni*.* Acidovorax caeni* is a facultative aerobic microorganism that is able to use carboxylic acids as carbon source [34]. Oxygen concentrations in the cathode gas phase were 0.6–3.5%. At these oxygen concentrations, aerobic bacteria that may act as oxygen scavengers have a physiological advantage compared to strict anaerobes. This physiological advantage is due to the higher reduction potential of oxygen reduction to water versus, for instance, carbon dioxide reduction to methane, respectively, 1.229 V versus NHE [35] and 0.169 V versus NHE [13] (at STP and 1 M or 1 atm for all components involved in the reaction).

For some of the bacterial phylotypes, their role in bioelectrochemical methane production remains unclear. For example, *Ottowia thiooxydans* is a facultative anaerobic microorganism that is able to use nitrate or nitrite for growth and able to oxidize thiosulfate and hydrogen to sulfate [36]. Also, *Petrimonas sulfuriphila *is a strictly anaerobic microorganism that is able to use sugars as carbon and energy source and able to reduce sulfur with hydrogen to sulfide [37]. 

In this study, insight is given into the role of the detected community members in methane production. While the identified 16 S rRNA sequences in our study most likely have similar substrate preferences as their closest relatives, this is not necessarily the case.

After 24 days, the biocathode produced only methane and no hydrogen. This start-up time is similar to reported start-up times for methane-producing biocathodes, that is, 28 days at −0.59 V cathode potential [8] and one month at −0.8 V cathode potential [13]. 33 days after initiating the methane-producing biocathode, the microbial community of the methane-producing biocathode was investigated (day 57 of the experiment). Although the current density was rather stable after start-up (0.6 ± 0.15 A/m^2^ projected cathode, Figure 2(a)) and only methane was detected, it remains unclear whether a stable microbial community was obtained.

### 3.4. Morphology and Distribution of Microorganisms at the Biocathode

**Figure 3:**
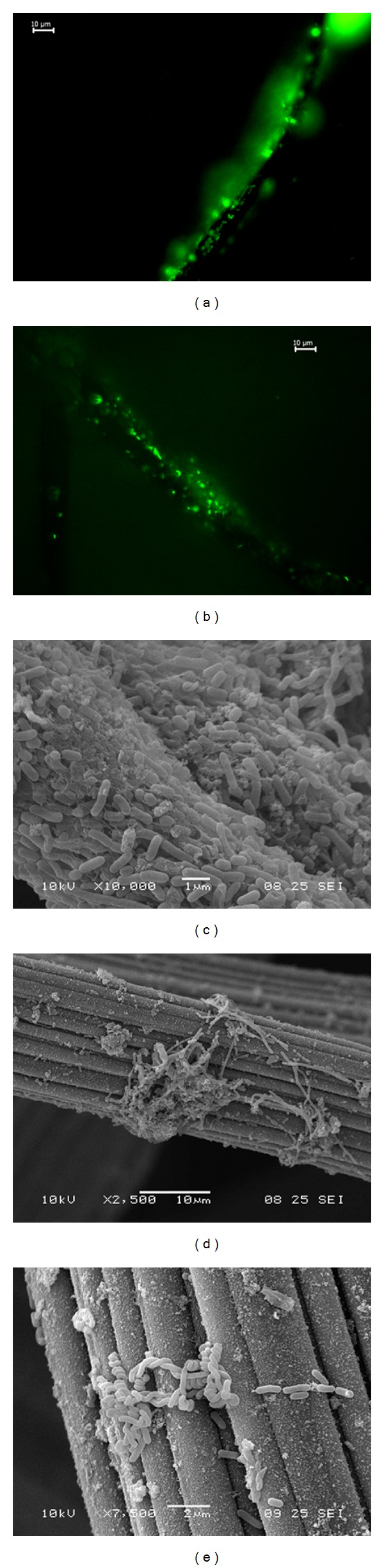
Fluorescence microscopy of the center of the biocathode revealed rod-shaped methanogens that were attached to the graphite felt fibers as single cells ((a) and (b)) or as microcolonies (a). Scanning electron microscopy (SEM) pictures of rod-shaped microorganisms at the center of the biocathode ((c), (d) and (e)). Part of the graphite felt was covered with a dense biofilm (c), while parts were only covered with clusters of microbial cells ((d) and (e)).

Microscopy techniques were used to give insight into the distribution of the microbial populations at the biocathode. With fluorescence microscopy, the presence of active methanogens in the biocathode can be revealed (Figures 3(a) and 3(b)). The observed microorganisms were rod-shaped 1–3 *μ*m long cells, with a few longer than 5 *μ*m. The cells were attached to the graphite felt as single cells or as microcolonies (Figures 3(a) and 3(b)). Whereas fluorescence microscopy is generally used to reveal the presence of active methanogens, it should be noted that it may also reveal the presence of reduced cytochromes of bacteria [38].

Scanning electron microscopy (SEM) revealed a variety of rod-shaped microorganisms at the biocathode, varying in their rod form (both straight rods and spiral-shaped rods were observed), the ability to form filaments, and their size (Figures 3(c), 3(d), and 3(e)). The length of the observed rods varied between <1 *μ*m and 10 *μ*m. The observed rod length was similar to that of the rod-shaped methanogenic archaea pictured by fluorescence microscopy (Figures 3(a) and 3(b)). No clear relationship was observed between the morphology and the location within the biocathode. Rod-shaped microorganisms varying in size from 1 to 5 *μ*m have also been observed in a previous mixed-culture biocathode that simultaneously produced acetate and methane [8].

SEM also revealed that part of the graphite felt was covered with a dense biofilm (Figure 3(c)), while other parts of the graphite felt were only covered with clusters of cells (Figures 3(d) and 3(e)). Both were observed at the low flow zone (where the cathode was located in a dead zone) and the high flow zone (where the cathode was in contact with the straight part of the flow channels). Accumulation of gasses (methane and hydrogen) inside and near the graphite felt electrode may have been an obstacle to attachment, thereby hindering biofilm formation. Another explanation might be that larger cell aggregates, consisting of both anaerobic methanogens and aerobic microorganisms acting as oxygen scavengers, are required to create the strict anoxic conditions needed by the methanogens. The absence of biomass on parts of the electrode could also be explained by a local excess of electron donor (e.g., hydrogen) near the electrode. As long as methanogens have access to electron donors further away from the electrode, there is no need to attach to the electrode and use the electrode as an electron donor.

### 3.5. Biomass Density at the Methane-Producing Biocathode

Based on the modified Hartree-Lowry analysis, it was found that the methane-producing biocathode contained on average 55.6 ± 11.9 g VSS/m^2^ projected cathode (*n* = 6 samples). This VSS density is in the range of reported VSS densities for bio-anodes, being 8–66 g VSS/m^2^ projected anode surface area [39]. This is the first study to report the VSS density for a methane-producing biocathode. The VSS density and thus the biomass density were similar at different locations of the biocathode: 57 and 68 g VSS/m^2^ projected cathode (*n* = 2 samples) where the catholyte entered the BES, 49 and 65 g VSS/m^2^ projected cathode (*n* = 2 samples) at the center of the biocathode, and 39 and 60 g VSS/m^2^ projected cathode (*n* = 2 samples) where the catholyte left the BES. No clear relationship was observed between biomass density and location at the biocathode. With the current experimental set-up, it could not be determined whether all biomass was active. The density of active biomass is an important parameter to improve conversion rates.

### 3.6. Microbial and Electrochemical Methods to Improve the Performance of a Methane-Producing Biocathode

Methane production rates can be improved by selecting for microorganisms that are involved in methane production or that create the optimal conditions for methane production. Selection strategies that could be used are (i) enrich the inoculum prior to inoculation, (ii) add pure cultures of *Methanobacterium palustre *and *Methanobacterium aarhusense* to the mixed-culture inoculum to give them a competitive advantage during start-up of the biocathode, (iii) adjust operational conditions to the optimum growth conditions for the preferred microorganisms, and (iv) stimulate the growth of synergistic bacteria that might be involved in bioelectrochemical methane production. Methods to enrich the inoculum prior to inoculation include growing the microbial community with an electrode or hydrogen as electron donor, either in batch experiments with multiple feeding cycles or by using the effluent of well-performing BES [8, 13, 16, 21, 40, 41]. This study shows that the phylotypes that are closely related to *Methanobacterium* species produce the preferred end product methane. Isolating the *Methanobacterium* species and testing them as a pure culture would be a first step towards verifying if these methanogens do in fact play a role in methane production at the biocathode as anticipated. Based on these results, enhancement of the number of cells of *Methanobacterium* species could be a strategy for increasing the methane production rate. Optimizing the operational conditions, such as cathode potential, pH, temperature, and mineral composition of the catholyte are known to positively affect BES performance [18]. Both *M. palustre* and *M. aarhusense* are mesophiles; *M. palustre *has its growth optimum at 37°C (pH 7) [26] and *M. aarhusense* at 45°C (pH 7.5–8) [27]. The temperature and pH used in this study were thus lower than the optimum conditions for *M. palustre* and *M. aarhusense*. Through adjusting the temperature and pH to the optimal growth conditions for methanogens, the electrochemically active bacteria as well as bacteria that act as oxygen scavengers, the methane production rates might increase further. Finally, this study revealed that bacteria, such as *Desulfovibrio putealis*, might be involved in bioelectrochemical methane production through production of hydrogen as intermediate. Synergistic relationships between bacteria and methanogenic archaea were also demonstrated by Cheng and coworkers, who reported that a mixed culture methane-producing biocathode dominated by *Methanobacterium palustre* performed much better than a pure culture biocathode with *Methanobacterium palustre* [13]. Stimulating the growth of synergistic bacteria, through either increasing their cell numbers during inoculation or by adjusting the operational conditions, might be viable strategies to further improve bioelectrochemical methane production.

Besides selecting for microorganisms that are involved in methane production, increasing the biomass density could further improve methane production rate. This study shows that the cathode was only partly covered with microorganisms. Likely, increasing the coverage of the cathode with biomass and increasing the biomass density will improve the performance of a methane-producing biocathode [18]. An excess of electron donor (e.g., hydrogen) near the electrode could have made it unnecessary for the methanogens to attach to the electrode. Biomass density can be increased by growing suspended methanogenic biomass on an inert carrier material that is kept in the catholyte. Additionally, biofilm formation could have been hindered due to accumulation of gasses (methane and hydrogen) inside and near the graphite felt electrode. In this study, the catholyte flowed alongside the cathode. Using a flow-through electrode results in improved mass transfer of gasses away from the electrode and substrates towards the electrode [42] and may consequently yield improved biofilm formation. Biomass coverage can also be increased by changing the cathode surface or catholyte composition for improved attachment of the microorganisms. Bacteria in natural systems usually have a net negative charge on the cell surface, resulting in electrostatic repulsion with negative charged surfaces, such as the cathode [43, 44]. Bacteria are, however, capable of adjusting their cell surface characteristics (charge and hydrophobicity) depending on the environment [44, 45]. Therefore, prolonged operation might improve bacterial adhesion. Bacterial adhesion could also be improved by changing the properties of the cathode surface, such as the hydrophobicity, and changing the catholyte composition, for example, pH and conductivity (e.g., [43, 46]). Another method to improve biomass density on the electrodes would be applying high shear [47]. In addition to improved mass transfer and more effective use of the cathode surface area [42], using a flow-through-electrode results in more compact biocathodes.

## 4. Conclusions

A methane-producing biocathode was obtained that produced methane at a maximum rate of 5.1 L CH_4_/m^2^ projected cathode per day (1.6 A/m^2^) at −0.7 V versus NHE cathode potential and 3.0 L CH_4_/m^2^ projected cathode per day (0.9 A/m^2^) at −0.6 V versus NHE cathode potential. The microbial community at the methane-producing biocathode was dominated by three phylotypes of archaea and six phylotypes of bacteria. The archaeal phylotypes were most closely related to *Methanobacterium palustre *and *Methanobacterium aarhusense*.  This study shows that, besides methanogenic archaea, bacteria may support methane production through production of hydrogen as intermediate or oxygen scavenging. 

## Figures and Tables

**Table 1:** Overview of the operational parameters, inoculum, and performance of methane-producing biocathodes that use CO_2_ as electron acceptor.

Microbial catalysts	Operational mode	Applied voltage^a^	Current density^b^	Methane production rate^c^	Reference
		(V (versus NHE))	(A/m^2^)	(L/m^2^ cathode per day)	
Defined enriched cultures—dominant microbe *Methanobacterium palustre *	Two-chamber, batch-fed	−0.8 V *E*_cat_	1.8	4.5	[13]
*Methanobacterium palustre* ATCC BAA-1077	Two-chamber, batch-fed	−0.8 V *E*_cat_	0.07	0.26	[13]
Undefined enriched cultures	Single-chamber, batch-fed	−0.807 V *E*_cell_	nr	nr	[15]
Undefined enriched cultures	Single-chamber, continuously-fed	−0.813 V *E*_cell_	nr	nr	[15]
Undefined enriched cultures	Two-chamber, batch-fed	−0.9 V *E*_cat_	2.9	9.2	[21]
Undefined enriched cultures	Single-chamber, continuously-fed	−0.9 V *E*_cell_	5.8	1.8	[16]
Undefined enriched cultures	Two-chamber, continuously-fed	−1.4 V *E*_cat_	4.1	8.7	[14]
Undefined mixed cultures	Two-chamber, continuously-fed	−0.7 V *E*_cat_	0.87	1.4	[17]
Undefined mixed cultures	Two-chamber, continuously-fed	−0.55 V *E*_cat_	0.21	0.12	[17]
Defined enriched cultures—dominant microbe *Methanobacterium *sp.	Two-chamber, batch-fed	−0.59 V *E*_cat_	nr	nr	[8]
Undefined mixed cultures	Single-chamber, batch-fed	−1.25 V *E*_cell_	nr	10	[48]
Undefined mixed cultures	Two-chamber, continuously-fed	−0.93 V *E*_cat_	0.10	0.24	[40]
Undefined enriched cultures	Two-chamber, batch-fed	−1.15 V *E*_cat_	15	24	[41]
*Methanobacterium thermoautotrophicus *	Single-chamber, batch-fed	−1.5 V *E*_cell_	nr	1.0	[49]
Defined mixed cultures—dominant microbe *Methanobacterium* sp.	Two-chamber, batch-fed	−0.7 V *E*_cat_	1.6	4.1	This study
Defined mixed cultures—dominant microbe *Methanobacterium* sp.	Two-chamber, batch-fed	−0.6 V *E*_cat_	0.9	2.3	This study

^a^Applied cathode potential (versus NHE) or applied cell voltage when the cathode potential was not reported.

^b^Calculated using the projected cathode surface area.

^c^Calculated at standard temperature and pressure (273.15 K and 1 atm) using the projected cathode surface area.

nr: not reported.

**Table 2:** Archaeal and bacterial 16S rRNA gene sequences of the methane-producing biocathode and their similarity with their closest cultured relative. The NCBI accession no. is given in parentheses.

	Clone number	Closest relative	Similarity (%)	Closest cultured relative	Similarity (%)	GenBank no.
Archaea	1379-1A8r	*Methanobacterium palustre* strain 21 (DQ649333.1) or strain Z2 (DQ649332.1)	99	*Methanobacterium palustre* strain DSM 3108 (NR_041713.1)	98	KC821542
	1379-1A19r	*Methanobacterium palustre* strain 21 (DQ649333.1) or strain Z2 (DQ649332.1)	99	*Methanobacterium palustre *strain DSM 3108 (NR_041713.1)	98	KC821543
	1379-1A1	Uncultured *Methanobacteriaceae* archaeon clone LrhA43 (AJ879024.1)	99	*Methanobacterium aarhusense* strain H2-LR (NR_042895.1)	96	KC821541

Bacteria	1379-1-24r	*Methylocystis* sp. WRS (AY007196.1)	99	*Methylocystis *sp. SC2 strain SC2 (NR_074220.1)	98	KC821549
	1379-1-23r	*Ottowia *sp. RB1-10B (EU882843.1) or *O. pentelensis* strain RB3-7 (EU518930.1)	99	*Acidovorax caeni* strain R-24608 (NR_042427.1) or *Hydrogenophaga caeni* strain EMB71 (NR_043769.1)	98	KC821548
	1379-1-6r	Uncultured delta proteobacterium clone MBNTA-bac1 (DQ205193.1)	98	*Desulfovibrio putealis *strain B7-43 (NR_029118.1)	97	KC821546
	1379-1-2	Uncultured bacterium clone YC50 (GU062460.1)	99	*Petrimonas sulfuriphila *strain BN3 (NR_042987.1)	96	KC821544
	1379-1-5r	Uncultured *Bacteroides* sp. clone 30-S33 (JX462549.1)	99	*Petrimonas sulfuriphila *strain BN3 (NR_042987.1)	95	KC821545
	1379-1-17	*Ottowia *sp. RB1-10B (EU882843.1) or* O. pentelensis* strain RB3-7 (EU518930.1)	99	*Ottowia thiooxydans* strain K11 (NR_029001.1)	95	KC821547
